# 2024年肺癌外科视角：创新与挑战

**DOI:** 10.3779/j.issn.1009-3419.2025.106.07

**Published:** 2025-03-20

**Authors:** Pengxu KONG, Xiaohan CHEN, Wang LV, Pinghui XIA, Luming WANG, Jian HU

**Affiliations:** 310003 杭州，浙江大学医学院附属第一医院普胸外科，全省医疗器械临床评价技术研究重点实验室; Department of Thoracic Surgery, The First Affiliated Hospital, Zhejiang University School of Medicine, Zhejiang Key Laboratory of Clinical Evaluation Technology for Medical Device, Hangzhou 310003, China

**Keywords:** 肺肿瘤, 外科, 创新进展, 手术方式, 全生命周期管理, 围术期治疗, Lung neoplasms, Surgery, Innovative advancements, Surgical approaches, Life-cycle management, Perioperative therapy

## Abstract

肺癌是全球范围内高发病率和高死亡率的恶性肿瘤之一。在我国肺癌的疾病负担尤为沉重，是最常见的癌症死亡原因。尽管医学快速进步，其发病率和死亡率仍居高不下，成为公共卫生领域亟待解决的重大难题。在精准医学快速发展的背景下，肺癌诊疗正在经历从传统单一治疗方式向多维度诊疗模式转变。本文系统回顾了2024年度肺癌外科领域的创新与挑战，旨在与同道学者们共同思考肺癌外科领域的未来发展道路，为提升患者生存质量与最终实现“治愈”目标提供思路。

## Abstract (en)

Lung cancer, a highly prevalent and deadly malignancy globally, poses a significant disease burden in China and is the leading cause of cancer death. Despite rapid advances in medicine, its incidence and mortality rates remain stubbornly high, making it a major challenge in public health. Against the backdrop of rapid progress in precision medicine, the paradigm of lung cancer treatment is shifting from single traditional therapy to multi-dimensional integration. This article comprehensively reviews the innovations and challenges in lung cancer surgery in 2024, aiming to explore the future development of surgical treatment with colleagues and to improve patients' quality of life and achieve the goal of "cure".

2024年肺癌流行病学调查呈现的“双高”（高发病率和高死亡率）对我国乃至全球公共卫生体系构成严峻挑战。肺癌的诊疗模式经历了巨大的变革，逐渐形成了局部治疗（手术、介入、放疗等）与全身治疗（化疗、靶向、免疫等）相融合的综合诊治模式。自2003年首个表皮生长因子受体（epidermal growth factor receptor, EGFR）靶向药物临床应用以来，在精准分子诊断的基础上，创新转化药物的临床应用不断取得重要突破，以总生存期（overall survival, OS）为目标的治疗理念，显著延长了患者的OS，使患者活得长、活得好，实现肺癌患者全生命周期的慢病化管理。免疫治疗的飞速发展，从病因学视角带来了诊疗效果的明显提升；免疫治疗的创新格局将为肺癌患者带来更高的治疗期待——治愈，尤其是可手术患者的围术期综合治疗。

2024年全球肺癌诊疗领域涌现出诸多创新成果转化。基于外科视角，本文聚焦2024年度重要临床研究及OS数据更新，以手术适应证为核心，系统回顾可手术肺癌患者外科优化诊疗模式，共同探索创新格局下的肺癌外科根治新理念、新策略、新实践。

## 1 “惰性肺癌”合理诊疗新进展

### 1.1 含磨玻璃结节（肺癌）规范化诊疗新思考

随着低剂量计算机断层扫描技术的广泛应用和公众健康意识的提高，越来越多的肺磨玻璃结节被发现。由于其生物学特性，磨玻璃结节肺癌的进展速度较慢，具有“惰性”发展和极少淋巴结或远处转移等特点，预后良好，可称之为“惰性肺癌”^[1]^。然而，其中部分仍存在缓慢进展的风险，需要适时干预治疗。因此“惰性肺癌”应以“不过度诊疗”与“不错过治疗机会”为原则，实现以临床手术适应证为核心的规范化诊疗。2024年，中国胸外科领域近百家临床中心及临床一线专家共同制定的《直径≤2 cm肺结节胸外科合理诊疗中国专家共识（2024）》（以下简称“共识”）正式发布^[2]^。

直径≤2 cm与>2 cm肺结节肺癌的外科治疗策略存在明显差异。胸外科医生在评估肺结节手术适应证时应以手术获益为治疗原则，避免过度诊疗。根据2024年共识，应根据结节的大小、成分、病灶部位等特征，采取多维度多因素综合诊疗模式，以“不过度不错过”为重要原则，实现“惰性肿瘤”的精准诊疗策略。对于≤2 cm实性肺癌或已完全实变的病灶，应特别关注并及时干预，避免“小病灶大转移”带来的不良后果。

医务工作者应遵循共识与指南采取合理的随访时间窗，并且结合患者的个体情况酌情分析，同时呼吁广大医务工作者，尤其是肺癌专科相关同行，加强早期肺癌的科普教育，在积极推广合理筛查的同时，建立正确的“惰性肿瘤”认识观，避免“谈癌色变”。

### 1.2 手术方式研究新进展

在实现手术患者身体状态评估的基础上，外科手术适应证的评估应实现三个维度：是否应该手术、手术的最佳时机和手术局部切除范围（术式选择）。

在评估早期非小细胞肺癌（non-small cell lung cancer, NSCLC）的最佳手术方式时，肿瘤的影像学特征和侵袭性预测至关重要。对于影像学上表现为实性病灶的NSCLC患者，肺段切除术与肺叶切除术的疗效差异成为关注焦点。2024年Lancet Respiratory Medicine刊发的一项事后分析^[3]^，基于JCOG0802/WJOG4607L研究，探讨了这两种手术方式的生存结局及复发模式。该项研究显示，影像学实性病灶的患者中，接受肺段切除术患者的5年OS高于肺叶切除术患者。然而，两组患者的局部复发率差异明显：肺段切除术的局部复发率约为肺叶切除术的2倍。这提示尽管肺段切除术在降低非肿瘤相关死亡风险方面具有优势，但在局部肿瘤控制方面仍存在不足，突显局部R0切除及手术质量控制的重要性。此外，患者的年龄和性别对手术效果具有显著影响，≥70岁患者接受肺段切除术的OS显著优于肺叶切除术，而对于<70岁患者，两种术式的OS差异不明显，但肺叶切除术的无复发生存期（relapse-free survival, RFS）更高；性别分析表明，男性患者在肺段切除术后的生存获益较为明显，而女性患者的生存结局在两种术式间差异较小。同时，研究还发现肺叶切除术患者中因非肿瘤相关疾病死亡的比例高于肺段切除术，这可能是前者OS率较低的主要原因。尽管如此，肺叶切除术在降低局部复发风险方面展现了更大的优势，特别是在年轻及女性患者中。因此对于实性病灶的NSCLC患者，手术方式的选择应综合考虑肿瘤特征、患者年龄及性别等因素，尤其是肿瘤的生物学活性等相关因素。肺段切除术虽然在提高OS率方面表现出色，但其局部复发率较高的特点需要通过术式改进或辅助治疗加以优化。这些结果为早期NSCLC的个体化手术决策提供了重要参考。

## 2 肺癌规范化手术模式——4S标准化围术期体系的实践与探索

肺癌外科治疗近年来经历了从传统经验导向逐步实现标准化、系统化模式的深刻转变。2024年浙江大学附属第一医院胸外科成功建立了以术前筛查、标准化手术方式及术后数字快速康复为核心的4S标准化围术期体系。“4S”包括自然腔道内镜手术（natural orifice translumenal endoscopic surgery, NOTES）、胸腔镜手术（video-assisted thoracoscopic surgery, VATS）、机器人辅助系统手术（robot-assisted thoracic surgery, RATS）和快速康复外科（enhanced recovery after surgery, ERAS）。这一创新性模式不仅提升了治疗效果，还为患者带来了全方位的获益，体现了现代医学对规范化和个体化诊疗融合的追求。项目组团队微创手术比例由2009年的20%增长至2022年的95%以上，微创手术中转为开胸手术的比例维持在0.5%以下，胸外科机器人手术量超2000例，位居全国前列。

近年来NOTES在肺癌早期诊断中展现出重要价值。这种技术通过优化软性与硬性内窥镜的应用，显著提升了微创诊断的精准性，并在推动国产内窥镜评价体系和技术标准化方面起到了积极作用。例如，4K高清与荧光技术的引入，为胸外科早期病灶的定位和诊治提供了可靠保障。这些创新突破不仅填补了技术空白，还推动了内镜技术在临床应用中的普及，使早期筛查更为精确，患者获益更加显著。然而，NOTES技术的操作难度较大，需要术者长时间经验的积累。随着技术的不断进步和术者经验的增加，其临床应用前景广阔，有望为更多患者提供微创、高效的诊疗方案。

如何在早期精准诊断基础上实现NOTES无痕手术技术的进一步发展，将是我们面临的挑战。VATS和RATS已成为肺癌微创治疗的重要手段。腔镜技术的不断迭代推动了从传统开胸到单孔胸腔镜的临床普及应用，通过减少手术切口和术式相关损伤，可显著降低术中风险和术后并发症。机器人手术系统与支气管镜手术机器系统的联合应用已成为2024年微创技术进展中的亮点，“内外夹攻，里应外合”将进一步提升机器人手术的精确性，为肺部多发病灶及复杂病例的外科干预提供更多更精准的治疗手段。尽管RATS在肺癌微创治疗中具有显著优势，如更高的稳定性和准确性，但其手术费用昂贵，且学习曲线较陡。此外，RATS在一些复杂手术中，如袖状肺叶切除术、新辅助治疗后的肺癌手术等，虽然具有一定的优势，但也存在一定的争议。因此，在临床应用中，应根据患者的具体情况和手术需求，合理选择手术方式，权衡新技术的优势和风险，以实现最佳的治疗效果。

ERAS在肺癌围术期管理中的实践为患者提供了全面而科学的干预策略。ERAS的核心是通过优化术前、术中及术后的管理环节，减少手术对患者生理和心理的负面影响，特别是在微创技术的支持下，“三无模式”即无气管插管、无胸腔闭式引流管、无导尿管的临床实践，使部分患者实现了日间手术（含周末手术）。这一模式显著缩短了住院时间、减少了术后并发症，并提升了患者的围术期康复体验。通过气道管理、营养支持和心理干预等多维度、一体化的多学科合作的深入推进，ERAS已成为提升围术期管理水平的标志性范例。

数字诊疗快速康复将进一步优化提升肺癌围术期管理。4S标准化围术期体系的推广，从理念到技术，已成为推动肺癌外科治疗现代化的重要内容。其核心理念是通过技术手段和科学管理，将手术的安全性、有效性与患者的舒适度结合，提升手术疗效，实现医疗资源的优化配置。4S模式以精准诊疗为基础，以患者康复为目标，为国内外医疗机构提供了可参考的先进范例。

数字诊疗与人工智能技术的进一步推动，将实现4S标准化围术期体系的升级和优化，有望形成胸外科日间手术及周末手术的常态化流程。围术期居家康复的创新数字化康复模式将是今后探索的重要内容之一。

## 3 肺癌外科全生命周期慢病化管理

随着肺癌的治疗模式不断改进与丰富，肺癌外科治疗逐步进入以“早期治愈”和“中晚期慢病化”为目标的综合治疗策略。对于肺癌外科而言，不能只关注围术期，更应着眼于患者的长期生存获益。除了传统的手术治疗，外科医生更需要关注肺癌的全生命周期管理。

### 3.1 可手术肺癌综合治疗新进展

#### 3.1.1 辅助治疗

在可手术的早期NSCLC患者中，围术期治疗已成为综合治疗的关键环节。2024年欧洲肿瘤内科年会上，备受关注的ALINA研究^[4]^进一步揭示了辅助靶向治疗在改善患者预后的重要作用，研究表明，对于间变性淋巴瘤激酶（anaplastic lymphoma kinase, ALK）阳性IB（≥4 cm）至IIIA期患者，阿来替尼辅助治疗可显著降低疾病复发或死亡风险，其无病生存期（disease-free survival, DFS）远优于传统含铂化疗。这一国际多中心III期研究再次强调了术后靶向治疗对延长早期肺癌患者OS的潜力。研究数据显示，无论淋巴结分期如何，阿来替尼在多种疾病特征的患者中均展现了良好的治疗效果，同时显著降低了中枢神经系统复发的风险。更为关键的是，阿来替尼在显著延长DFS的同时，安全性良好，不良事件发生率和停药率均低于化疗组，进一步凸显了其临床价值。这一研究不仅证实了靶向辅助治疗在延长生存期方面的优势，也为ALK阳性早期肺癌患者提供了更具前景的治疗策略，推动了围术期综合治疗模式的进一步优化。ALINA研究成果的发布，为可手术肺癌治疗带来了新的希望，有望助力实现外科可手术患者的“治愈”目标。

#### 3.1.2 新辅助治疗

NSCLC的围术期治疗取得了突破性的进展。部分NSCLC在靶向治疗、免疫治疗的作用下，不仅实现了病理完全缓解（pathological complete response, pCR），更明确了远期事件——无事件生存期（event-free survival, EFS）的获益，趋向实现OS的转化^[5,6]^。

在2024年世界肺癌大会上AEGEAN研究的第二次中期分析更新结果^[7]^显示，度伐利尤单抗组的DFS显著优于安慰剂组，进一步证实了免疫治疗在控制术后复发方面的优势。尽管OS数据尚未成熟（成熟度为35.3%），但已有证据显示度伐利尤单抗组的OS有潜在优势，两组的中位OS分别为未达到和53.2个月[风险比（hazard ratio, HR）=0.89]，表明度伐利尤单抗组的死亡风险相对更低。这一积极趋势为围术期免疫治疗可能带来的长期生存优势提供了关键证据。围术期治疗已成为综合治疗的核心环节之一，通过提高手术完全切除率（R0切除）、减小手术范围以及控制潜在微转移，新辅助治疗将为患者提供更加显著的生存获益。

2024年，围术期免疫治疗的“夹心饼干”全程模式备受关注。“夹心饼干”模式通过术前新辅助免疫治疗激活特异性T淋巴细胞、缩瘤降期，并发挥原位疫苗效应；术后辅助免疫治疗则清除残留微转移病灶、逆转免疫抑制。此模式实现了围术期全程免疫激活，最大限度地发挥了抗肿瘤作用。尽管尚缺乏与传统新辅助治疗直接对比的研究数据，但已有研究^[8]^显示，这种模式在不同患者群体中展现出较明显的优势。在新辅助靶向治疗领域，奥希替尼针对EGFR突变的早期NSCLC患者显示了良好的安全性和一定疗效，但主要病理缓解率（major pathological response, MPR）尚未达到预期。免疫治疗方面，多项国际研究如CheckMate 77T^[9]^、KEYNOTE-671^[6]^以及中国的Neotorch^[10]^和RATIONALE-315研究^[11]^均表明，新辅助和辅助免疫联合化疗能够显著提高pCR和EFS，并改善长期生存预后。特瑞普利单抗和替雷利珠单抗作为国产免疫治疗药物，在国内研究^[10,12]^中也展现出显著疗效，为术前及术后的免疫治疗提供了重要的数据支持。

在早期可手术肺癌的治疗中，外科手术依然是治疗的核心，特别是在pCR作为新辅助治疗效果的重要指标的背景下，外科手术的质量控制愈发重要。手术适应证评估应思考：手术的最佳时机（新辅助后）、局部降期与微小残留病灶（minimal residual disease, MRD）的控制以及手术局部切除范围优化（保功能、保器官）。尽管新辅助治疗已经显著提高了pCR率，这一指标是否能够转化为更长的EFS和OS获益，仍然是当前临床治疗研究中的关键问题。现有研究^[13]^显示，pCR的获得通常与更好的长期预后有关，但是获得pCR的患者是否都能实现长期的OS转化，仍然存在不确定性。医务工作者应严格遵守外科R0切除原则，但是否可以减少pCR患者的切除范围、切除范围与局部复发概率如何权衡、手术方式是否应该根据新辅助治疗后的分期进行选择，这些将是未来研究的重点。

### 3.2 晚期肺癌外科治疗新探索

#### 3.2.1 以OS为治疗标准的不可手术肺癌转化治疗后的外科治疗

外科手术的最终目标是为了实现R0切除，局部根治。在全身治疗（靶向、免疫联合化疗）取得疗效重大突破的背景下，外科治疗的适应证也随着创新格局的改变而改变。全身诱导治疗对不可手术、IIIA-IIIB期部分患者有望实现局部手术切除的转化。如何界定不可切除与可切除的界限将再次面临挑战。不可切除转化为可切除患者是否能在局部根治性R0切除的基础上实现OS的转化，在相关学科如乳腺癌、结直肠癌等均已有临床研究及临床数据支持^[14]^。PACIFIC研究^[15]^带来了III期不可切除患者在同步放化疗联合免疫维持治疗下5年OS的明显提升。而转化治疗后的手术R0切除联合维持治疗是否能带来更好的OS预期，是否能在PACIFIC研究的基础上带来更长的生存获益，将是我们面临的挑战，因此有必要进行进一步的临床研究。尤其是目前与多学科同道开展III期的新辅助研究当中，已包含了IIIA与IIIB期不可手术的病例，尽管病例数偏少，但其亚组分析已提示转化治疗带来的相关优势，如吴一龙团队^[16]^报道了不可手术肺癌患者接受新辅助联合化疗后，手术治疗在25%的患者中是可行的，并且与非手术患者相比有更好的生存结果。未来不可手术肺癌转化模式是否会改变应进一步探索。

#### 3.2.2 以生存获益为目标的晚期寡转移肺癌的外科巩固治疗

IV期肿瘤以往无法实现外科手术治疗，但对IV期寡转移病例仍有外科治疗后获益的相关总结。在靶向精准治疗与免疫治疗取得重大突破的新格局下，IV期寡转移患者已实现了生存获益明显提升。与瘤共存，活得长、活得好是目前晚期IV期患者的治疗标准。但如何活得更长、活得更好，进一步探索肿瘤耐药及禁止期状态下的机制，进一步提升生存获益，将是我们面临的挑战。随着MRD检测技术的发展，如何实现循环肿瘤DNA（circulating tumor DNA, ctDNA）和细胞游离DNA（cell-free DNA, cfDNA）甲基化等肿瘤微转移状态的评估与精准判读，以及对药物假期等创新策略的研究探索，将是我们面临的重要课题。

肺癌外科手术局部巩固治疗，是以生存获益为总目标的晚期寡转移肺癌治疗策略的重要挑战。美国安德森肿瘤中心2016年和2019年对晚期转移肺癌寡转移患者的临床研究^[17,18]^，证明了全身综合治疗基础上的局部巩固治疗（手术/放疗）获得了DFS、OS的明确获益，强调了IV期寡转移患者全身治疗窗口期精准评估局部治疗的重要性。结合ctDNA相关动态监测，通过手术适应证的精准评估，将为IV期寡转移患者带来更优化的局部手术巩固治疗^[19]^。IV期寡转移肺癌全身治疗窗口期的外科手术巩固治疗，是否能实现局部及全身综合治疗的更大生存获益、手术标本是否能为后续治疗带来更深入的机制研究及药物转化、能否为全身治疗带来更佳的诊疗策略，如升阶治疗、降阶治疗的选择、药物假期的合理时长，目前多项相关的临床研究正在进行当中，如浙江大学附属第一医院正在探索寡转移患者程序性死亡受体1（programmed death-1, PD-1）单抗联合化疗一线治疗后行局部巩固治疗，有望为患者提供新治疗方案。

新时代、新格局、新策略，基础研究转化与临床研究相互推动，循证证据与回顾性大数据融合，多学科同道的共同努力，将为可手术肺癌患者带来全新治疗模式的升级，助力实现治愈目标及全生命周期慢病化管理。
